# Editorial: Advances in mathematical and computational oncology, volume III

**DOI:** 10.3389/fonc.2023.1282882

**Published:** 2023-09-25

**Authors:** George Bebis, Mamoru Kato, Mohammad Kohandel, Kathleen Wilkie, Dinler A. Antunes, Ken Chen, Jinzhuang Dou

**Affiliations:** ^1^ Department of Computer Science and Engineering, University of Nevada, Reno, NV, United States; ^2^ Division of Bioinformatics, Research Institute, National Cancer Center Japan, Tokyo, Japan; ^3^ Department of Applied Mathematics, University of Waterloo, Waterloo, ON, Canada; ^4^ Department of Mathematics, Ryerson University, Toronto, ON, Canada; ^5^ Department of Biology and Biochemistry, University of Houston, Houston, TX, United States; ^6^ Department of Bioinformatics and Computational Biology, Division of Basic Science Research, The University of Texas MD Anderson Cancer Center, Houston, TX, United States

**Keywords:** mathematical oncology, computational oncology, machine learning, mathematical modeling, computational modeling

Despite significant advances in the understanding of the principal mechanisms leading to various cancer types, less progress has been made toward developing patient-specific treatments. Advanced mathematical and computational models could play a significant role in examining the most effective patient-specific therapies. Tumors, for example, undergo dynamic spatio-temporal changes, both during their progression and in response to therapies. Multiscale advanced mathematical and computational models could provide the tools to make therapeutic strategies adaptable enough and to address the emerging targets. Similarly, understanding the interrelationship amongst complex biological processes requires analyzing very large databases of cellular pathways. This Research Topic includes contributions to the state of the art and practice in mathematical and computational oncology addressing some of the challenges and difficulties in this field, as well as prototypes, systems, tools, and techniques.

Atsou et al. provided partial differential equations to represent interactions between immune and tumor cells. Introducing the equilibrium state between cancer cell proliferation and the elimination by immune cells, they invented a numerical method that determines the tumor size at the equilibrium from biological parameters. A sensitivity analysis was performed to find that the elimination rate of tumor cells by immune cells and their combinations with other parameters were the most influential factors. This suggests that the most effective strategy in immunotherapies is to act on the immune system rather than the tumor itself.

Chen et al. describes a study that investigated the tumor microenvironment (TME) characterization in gastric cancer (GC) patients and its association with recurrence, survival, and therapeutic response. The study used gene-expression data and clinical annotations from twelve cohorts of GC patients and evaluated the TME characteristics using three computational algorithms. The study developed a TME-classifier, a TME-cluster, and a TME-based risk score to predict recurrence, survival, and response to chemotherapy and immunotherapy. It found that TME characterization was significantly associated with these outcomes and identified subgroups of patients who benefited from different treatments.

Yan et al. provide a timely to the field review on the application of network control methods in personalized cancer genomics. The authors have focused on methods that can be applied to one or few samples from an individual patient, for personalized medicine. Directions for future research are also highlighted providing a useful resource for researchers in the field.

RNA methylation (m6A) plays a significant role in numerous crucial physiological processes. Huang et al. analyze multi-omics data from 568 soft tissue sarcoma (STS) patients to investigate the relationship between m6A mRNA methylation, metabolic pathways, tumor microenvironment (TME), and patient prognosis. Using machine learning algorithms, they identify two distinct subtypes based on m6A-related metabolism and establish a scoring system (m6A-metabolic Score) to predict patient prognosis and immunotherapeutic responses. Additionally, they identify 11 m6A-related metabolic pathways associated with STS prognosis and demonstrate differences in TME characteristics and stemness features between the subtypes. This study provides insights into the potential for targeting m6A-related metabolism in STS treatment and guidance for personalized immunotherapy.

Chang et al. describes the role of DDOST (a protein involved in N-glycosylation) in gliomas, a type of brain tumor. The authors found that DDOST was overexpressed in gliomas and correlated with poor prognosis, tumor grade, IDH status, 1p19q status and MGMT methylation status. They showed that DDOST was associated with the immune microenvironment of gliomas and negatively related to tumor-infiltrating B cells and CD4+ T cells and positively related to CAFs and tumor-associated macrophages. They suggested that DDOST could be an important biomarker for diagnosing and treating gliomas.

Immunotherapy has provided new treatment options for cancer patients, with particular success against melanoma and lung cancer (1). However, response to treatment can vary dramatically, and there are no reliable biomarkers to predict response in a personalized manner. In this context, Yao et al. aimed at exploring novel immunological classifications associated with immunotherapy response through the Single-sample Gene Set Enrichment Analysis (ssGSEA) algorithm. They established a novel score to evaluate the immune-related risk (IRS). This IRS model was linked to prognosis and other tumor characteristics, predicting overall survival and immunotherapy response in non-small cell lung cancer patients.

Chen et al. examined the gene expression patterns of costimulatory molecule genes in patients with stomach adenocarcinoma (STAD) and developed a predictive signature to aid in therapy selection and outcome prediction. The authors conducted the first complete costimulatory molecular analysis in patients with STAD using 60 costimulatory family genes from prior research. They identified nine costimulatory molecular gene pairs (CMGPs) with prognostic value and developed a costimulatory molecule-related prognostic signature that performed well in an external dataset. The signature was proven to be a risk factor independent of the clinical characteristics. A further connection between the signature and immunotherapy response was discovered, suggesting that high-risk patients may have a better prognosis for immunotherapy.

The action of micro-RNAs (miRs) and their target genes during clear cell renal carcinoma (ccRC) progression was investigated by Zamora-Fuentes et al. To identify miRs that may have different roles during cancer progression, they developed a methodology for constructing miR-gene co-expression networks for each progression stage of ccRC, as well as for adjacent-normal renal tissue. Using these networks, they were able to observe those miR-gene interactions that are shared and unique for all the progression stages. The main finding of their study is that although miR-217 is differentially expressed in all contrasts, its targets were different depending on the ccRC stage.

There is a trend in implementing artificial intelligence methods on predicting cancer immunotherapy. Li et al. employed an auto-encoder workflow to extract features from binary genotype data for cancer prognosis prediction. The compressed transformation could better improve the model’s original predictive performance and might avoid an overfitting problem due to the high dimensional data. Similarly, Shen et al. has demonstrated the efficiency of immunotherapy prediction based on artificial intelligence neural network. Their study enrolled 289 lung squamous carcinoma patients who received immunotherapy at Beijing Chest Hospital. With clinic features fed in the training model, the predicted disease control rate (DCR) can reach 0.95. In the work by Zhu et al. the researchers further characterize the tumor microenvironment (TME) of colorectal cancer based on cuproptosis-related molecular pattern. They derived the cuproptosis-related molecular patterns from 1,274 colorectal cancer samples. Such cuproptosis-related features are likely to strengthen our understanding of TME.

Zabor et al. proposes three randomized designs for early phase biomarker-guided oncology clinical trials. The designs use the optimal efficiency predictive probability method to monitor multiple biomarker subpopulations for futility. A simulation study results suggest that potentially smaller phase II trials can be designed using randomization and futility stopping to efficiently obtain more information about both the treatment and control groups prior to phase III study.

Yang et al. use mathematical modeling to explore the emergence of chemoresistance in a breast cancer cell line (MCF-7) treated with doxorubicin. Starting with logistic growth, they assume that a new subpopulation of irreversibly damaged cells is created after each dose. They fit their model to *in vitro* data and explore the dependence of model parameters on increasing drug dose, inter-treatment interval, and number of doses. This work suggests that longer delays between doses may promote chemoresistance.

A visual nomogram for cancer-specific survival and overall survival is proposed by Wang et al. Using lung neuroendocrine cancer SEER 18 data, they compute the log of the ratio of the number of positive lymph nodes to the number of dissected, non-positive lymph nodes. They then use multivariable Cox regression analysis to construct nomograms to predict survival, and show that its prognostic ability is an improvement over the existing tumor-node-metastasis staging system.

Zhou et al. introduces a framework for conducting landmark mediation survival analyses that incorporate longitudinal assessment of tumor burden in clinical cancer trials. Zhou et al. compare the predictive performance of different tumor response characterizations, including conventional RECIST criteria and longitudinal tumor burden assessments using functional principal component (FPC) scores and integrated response measures. The framework is applied to two colorectal cancer trials, comparing survival prediction with and without longitudinal analysis. The findings show that longitudinal models utilizing FPC scores yield higher predictive accuracy, particularly when tumor burden exhibits U-shaped trends. In cases without U-shaped trends, binary objective response provides limited information. The study highlights the importance of incorporating longitudinal tumor burden data in treatment survival mediation analysis and suggests the practicality of FPC scores for capturing these patterns.

In recent years, natural killer (NK) cells have also emerged as an alternative to T-cell-based immunotherapy, as in cases of secondary resistance to checkpoint blockade therapy (2). NK cells are known to play a protective role in colorectal cancer (CRC), but most patients show limited intra-tumoral NK cell infiltration. In their new study, Shembrey et al. identified a new NK cell-specific gene signature to predict recurrence in colorectal cancer patients. By prioritising genes based on NK-specificity rather than expression level, they increased precision when determining the NK-specific contribution. High NK score was associated with several clinically useful molecular parameters, as well as with improved survival outcomes in CRC patients.

Developing noninvasive markers to assess the tumor microenvironment signature is of great significance in clinical practice. In the study by Li et al., the correlation between radiomics and immune microenvironment was determined using the preoperative PET/CT imaging data and the immunohistochemical results of the immune microenvironment of the pathological tissue after the operation. The main result of this study is that the radiomics score is related to the survival of patients and the benefit of adjuvant chemotherapy.

Some personalized forms of cancer immunotherapy rely on immunoinformatics tools, such as AI-based predictors of peptide-binding to Human Leukocyte Antigen (HLA) receptors (3). Although powerful, these methods can suffer from biases introduced by the limitations of available training sets, as recently demonstrated by Solanki et al. Their analysis showed that the allele-specific tool NetMHC-4.0 has a statistically significant bias towards predicting highly hydrophobic peptides as strong binders to two HLA alleles with differing hydrophobicity requirements. On the other hand, the pan-allele tool NetMHCpan-4.1 provides more reliable predictions, potentially due to the inclusion of immunopeptidomics on the training data.

Hepatocellular carcinoma (HCC) is a common and deadly cancer. Mou et al. focused on lipid metabolism-related genes differentially expressed between primary and metastatic HCC in single-cell RNA sequencing data. From these genes, they further selected 8 genes with a machine learning technique in TCGA bulk-cell RNA sequencing data and constructed a Cox regression model with the selected genes. Surprisingly, this model was validated with ICGC data. Clinical utility was shown in their monogram and decision curve analysis. This study represents an important step forward in the fight against the devastating disease.

Partin et al. presents a novel approach to improve drug response prediction in patient-derived xenografts (PDXs) using neural networks. The authors propose to combine drug descriptors, gene expressions and histology images as inputs for a multimodal neural network (MM-Net) and to augment the data by homogenizing drug representations and doubling drug-pair samples. They show that their method outperforms unimodal neural networks and baselines in terms of prediction performance. The article demonstrates the potential of data augmentation and multimodal learning for advancing cancer research with PDXs.

Agent-based models (ABMs) are effective tools for capturing intratumoral heterogeneity and integrating various scales of tumor dynamics. However, the computational costs of ABMs become expensive when simulating large cell populations, making parameter exploration and calibration challenging. Jain et al. present a novel method called Surrogate Modeling for Reconstructing Parameter Surfaces (SMoRe ParS), which uses surrogate models as intermediaries between ABM inputs and experimental data. The authors demonstrate the effectiveness of SMoRe ParS using an ABM of 3D vascular tumor growth as the computational model and utilized data obtained from tumor xenograft growth experiments as the real-world data. This application is an illustrative example of how SMoRe ParS can effectively connect the outputs of computational models with empirical data.

Deutscher et al. present a hybrid cellular automaton model describing the cancerization of an area due to carcinogen exposure. They focus on tobacco and alcohol exposure and implement a multi-layer perceptron to infer the resulting gene-related changes potentially leading to cancer development. With this model, they explore mutation and phenotypic evolution due to exposure, the effect of tumor excision, and the heterogeneity of clones in emerging tumors. Their results suggest that partial excision can lead to more aggressive recurrence and that tumors mainly form through polyclonality.

Partin et al. present a comprehensive review of machine learning methods for drug response prediction mainly from omics data, with strong attention to deep learning. This review extensively covers deep learning structures such as CNN, graph neural networks, and transformers, as well as learning schemes such as autoencoders, transfer learning, and multi-task learning. Additionally, the authors provide all other aspects of the method development, including data preparation, cancer models, measures of response, representations of drug compounds, cross-validation schemes, baseline models, and useful development practices. This review will be an essential guide for ones who develop and use machine learning for drug response prediction.

A machine learning methodology for the identification of multi-gene predictive biomarkers for targeted cancer drugs was developed by Shin et al. The methodology was applied to identify highly predictive biomarker panels for Hsp90-targeted treatment in prostate cancer from patient-derived proteomic data. The authors were able to identify 5-protein panel biomarker that can predict cancer drug sensitivity. This is a promising approach, however, additional validation on different tumor types and cancer drugs is needed to further establish the usefulness of the method.

Local and regional recurrence of cancer after surgery is a major problem in cancer management, and the means to identify patients who are more likely to have a shorter recurrence are still lacking (4, 5). To address this issue, Abubakar et al. developed a mathematical model of cancer development using Moran and branching processes. The model is fit to historical data on disease-free survival in 27 difference cancer types and is used to estimate the cell turnover rate per month, relative fitness of pre-malignant cells, growth rate and death rate of cancer cells in each cancer type.

## Author contributions

GB: Writing – original draft. MK: Writing – original draft. MK: Writing – original draft. KW: Writing – original draft. KC: Writing – original draft. DA: Writing – original draft. JD: Writing – original draft.
